# Nerve conduction during acute blood-flow restriction with and without low-intensity exercise Nerve conduction and blood-flow restriction

**DOI:** 10.1038/s41598-020-64379-5

**Published:** 2020-04-30

**Authors:** Goncalo V. Mendonca, Miguel Mouro, Carolina Vila-Chã, Pedro Pezarat-Correia

**Affiliations:** 1 Neuromuscular Research Lab, Faculdade de Motricidade Humana, Universidade de Lisboa, Estrada da Costa, 1499-002 Cruz Quebrada, Dafundo Portugal; 2 CIPER, Faculdade de Motricidade Humana, Universidade de Lisboa, Estrada da Costa, 1499-002 Cruz Quebrada, Dafundo Portugal; 3 Polytechnic Institute of Guarda, Av. Dr. Francisco Sá Carneiro, n. 50, Guarda, 6300-559 Portugal; 4 Research Center in Sports Sciences, Health and Human Development (CIDESD), Vila-Real, Portugal

**Keywords:** Neural circuits, Neurophysiology

## Abstract

Despite being apparently safe for most individuals, the impact of low intensity (LI) blood-flow restricted (BFR) exercise on nerve function and integrity is still obscure. We explored whether BFR (with and without exercise) alters the properties of nerve conduction measured at the level of the restricted limb. Thirteen healthy, young men (22.0 ± 1.7 years) were included in this study. Arterial occlusion pressure was taken at rest. Soleus M- and H-recruitment curves were constructed for all participants. H-wave latencies and amplitudes were obtained in three testing conditions (non-BFR vs. 60 vs. 80% BFR) at four different time points: [#1] non-restricted baseline, [#2] time control either with or without BFR, [#3] non-restricted pre-exercise, [#4] LI exercise either with or without BFR. Nerve conduction was estimated using the difference between the latency of H and M wave. BFR did not affect H-wave amplitude, either with or without exercise. The changes in the difference between H- and M-wave latency of over time were similar between all conditions (condition-by-time interaction: F = 0.7, p = 0.47). In conclusion, our data indicate that performing LI exercise with BFR, set at 60 or 80% BFR, does not exert a negative impact on sciatic-tibial nerve function. Thus, from a neurological standpoint, we provide preliminary evidence that LI BFR exercise may be regarded as a safe mode of resistance training in healthy young men.

## Introduction

There is compelling evidence that blood flow restriction (BFR) during low-intensity (LI) exercise induces similar neuromuscular adaptations (e.g. gains in muscle hypertrophy, strength and work capacity) as high-intensity training (HI), but using considerable less load^1–4^. Despite being apparently safe for most individuals, regardless of age and training status^5^, the impact of LI BFR exercise on nerve function and integrity still not well understood^6,7^. Survey studies, exploring the adverse effects of BFR, observed numbness as a transient side effect in 1.6 to 18.5% of the training sessions^7–9^. On the other hand, in some experimental studies, the majority of participants felt numbness during ischemic exercise^10^. Such discrepancy on the prevalence of numbness is likely explained by different LI BFR methodologies. Although cuff pressure, size and shape have been identified as key factors for safety and efficacy of BFR training^6^, there is wide variation of cuff type and pressure^5^ used between studies and among practitioners^7^. Higher pressure and/or wider cuffs increase the risk of nerve ischemia and reduce nerve conduction velocity, thus augmenting numbness sensations. These adverse effects are transient and usually do not result in long-term side effects, at least in healthy persons^11,12^. However, more research is required to establish whether this exercise approach impairs peripheral nerve function acutely^6^. This is particularly important when taking into consideration that LI BFR has been used for therapeutic purposes in patients with disturbed peripheral nerve function (both sensory and motor)^9^.

Data from past research demonstrate that the pressure applied to a peripheral nerve inhibits energy-dependent processes, namely the Na^+^/K^+^ pump. Such alterations lead to the depolarization of sensory and motor axons, resulting in a biphasic excitability response (i.e. first there is an increase and then a reduction in nerve excitability)^13–15^. Although both types of peripheral axons are sensitive to ischemic conditions, sensory axons show faster inexcitability during ischemia and therefore are particularly vulnerable to its effects^14,16^. In addition, nerve compression and ischemia are well known to prolong nerve latencies and reduce the amplitude of compound motor action potentials^15^. Such changes might modulate the excitability of spinal circuitry, thus leading to distinct neurophysiological and/or motor control adaptations.

In humans, H-reflex methodology is a useful tool to investigate both sensory and motor nerve conduction velocity^17^. It also allows further insight into the efficacy of synaptic transmission because, after being triggered, the stimulus travels in afferent (Ia sensory) fibers to the motoneuron (MN) pool of the corresponding muscle and, from then on, to the efferent (motor) fibers^18,19^. The afferent (sensory) portion of the H reflex starts at the point of electric stimulation of a mixed nerve and results in afferent action potentials that travel along the Ia afferent fibers towards the spinal cord. At the spinal level, Ia axons exert a strong monosynaptic excitatory connection with the MN of the homonymous pool^19^. Consequently, if the Ia volley is strong enough to elicit a compound excitatory postsynaptic potential (EPSP), the low-threshold MN will generate action potentials directed towards the neuromuscular junction and traveling along the efferent fibers. Ultimately, this will generate muscle-fiber action potentials that can be detected by electromyography (EMG) (H reflex). In addition, an increment of the intensity of stimulation at the level of the peripheral nerve causes direct activation of efferent fibers. Thus, a compound muscle action potential can also be recorded over the muscle of interest via EMG recordings (M wave)^20^. M-wave assessment can provide relevant information on peripheral properties of the neuromuscular system, without the direct involvement of the central nervous system (e.g. excitability of the muscle membrane and neuromuscular junction)^21^. Therefore, the analysis of evoked potentials elicited by peripheral nerve stimulation might allow a better understanding of the effect of LI BFR on peripheral nerve properties and MN net excitability. Surprisingly, few studies have investigated the impact of BFR on H reflex and M wave. Acute ischemia seems to induce a decline in H-reflex and M-wave thresholds with longer lasting effect for the H reflex decrease^22,23^. In addition Zakutansky et al., observed a decline in H_max_/M_max_ ratio, indicating that acute ischemia has differential effects on sensory nerve propagation and synapse transmission^23^. However longitudinal studies did not observed any significant alterations of the H-reflex and M-wave parameters^12^.

Ischemic conditions have been induced by different magnitudes of values of cuff pressure and sizes typically used within the context of LI BFR exercise (>180 mmHg)^15,24^. However, current research on LI BFR exercise acknowledges the importance of prescribing an individualized cuff pressure and further recommends setting a relative BFR pressure based on a percentage of the individual arterial occlusion pressure (AOP)^25^. This approach is recommended because it allows for the application of a pressure specific to the person and cuff type being used in each case. Yet, it is unknown whether this is compatible with preserved nerve conduction in circumstances where BFR is adjusted to the recommended training pressures (i.e. between 40–80% BFR in the lower limb)^25–29^.

Considering all these aspects, this study aimed at exploring whether BFR alters the properties of nerve conduction measured at the level of the restricted limb. We also sought to determine if the impact of BFR on nerve conduction might depend on the magnitude of BFR (60 vs. 80% BFR). Additionally, we tested if the impact of BFR on nerve conduction properties changed in the context of LI exercise. We hypothesized that BFR set at 80% AOP would disturb nerve conduction more profoundly than at 60%. In addition, based on previous data reporting occasional numbness in response to acute LI BFR exercise^30^, we hypothesized that nerve conduction would be further disturbed with LI BFR exercise vs. BFR alone at similar levels of relative BFR.

## Methods

### Participants

Thirteen healthy, young men (age: 22.0 ± 1.7 years, height: 175.2 ± 3.9 cm, body mass: 68.4 ± 5.4 kg and body mass index: 22.3 ± 1.5 kg/m^2^) were included in this study. Participants were recruited from the Faculty surroundings via word-of-mouth. All participants were experienced exercisers and they all were similarly active, accumulating 9 h of physical activity as part of their academic work. The risks of participation were carefully explained to all participants. Informed consent was obtained from all individual participants included in the study. At study entry, participants were required to complete a health-screening questionnaire and a degree of footedness questionnaire^31^. Only right-footed participants, exhibiting strong right dominance (score ≥ +20), were included in this study. Participants were also required to complete a health-screening questionnaire. They were all non-obese, normotensive and free from any cardiovascular, metabolic, respiratory and/or orthopedic disease. None of the participants reported any symptoms of numbness, tingling or distal motor weakness. To ensure greater reproducibility of arterial pressure values between testing days (which is known to affect BFR^32^), each participant was requested to abstain from heavy exercise for at least 48 h before testing and to have nothing to eat or drink from midnight until the testing session on the subsequent morning.

### Study design

The experiments were performed during the morning period, between 07:00 and 11:00 h, in a laboratory at a temperature between 22–24 °C and a relative humidity between 44–56%. In a randomized and counterbalanced manner, all participants were tested on three non-consecutive days within a one-week period (non-BFR vs. 60 vs. 80% BFR). Participants were familiarized with the experimental tests and protocol one week before testing. Familiarization consisted of one session that served the purpose of ensuring adaptation to all testing procedures (including AOP measurement, metronomic pacing and LI BFR exercise). Body mass and height were taken with the participants wearing lightweight clothes and no shoes. Height was obtained by using a stadiometer to the nearest 0.5 cm. Body mass was measured on a digital scale to the nearest 0.01 kg (TANITA® BF-350 body composition analyzer, Arlington Heights, IL). Body mass index was then calculated by dividing the participants’ body mass in kilograms by the square of their height in meters. Resting blood pressure (BP) was also obtained post-5 min of seated rest after completing the anthropometric measurements using an automatic BP monitor, in duplicate (Tango SunTech Medical Morrisville, NC). For analysis, the average of the two resting BP values obtained at each time point was used. If the values were not within 5 mmHg, a third measurement was taken and the two closest values were averaged and used for analysis.

**Figure 1:**
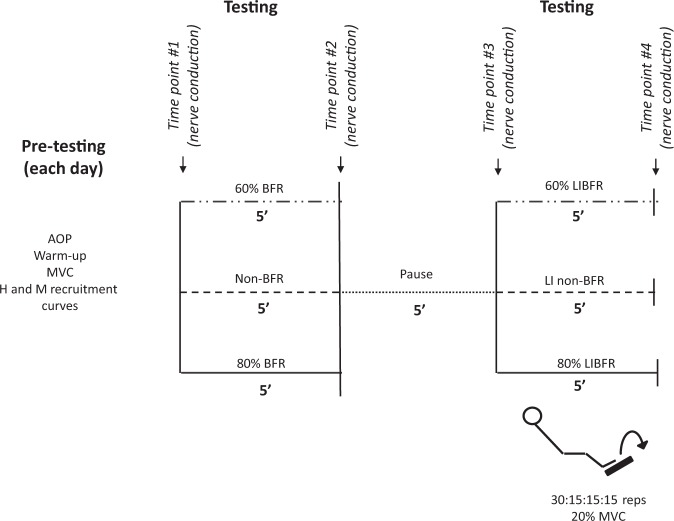
Schematic representation of the experimental design. The arrows represent the time points at which the amplitude and latency of H wave was obtained in each condition (non-blood-flow restriction [BFR], 60 and 80% BFR). Pre-testing measures were taken each day and included the following parameters: arterial occlusion pressure (AOP), maximal voluntary contraction (MVC), H and M-recruitment curves.

Each BFR testing session began with the determination of resting AOP in the dominant lower limb. Then, in all sessions, participants performed a general warm-up consisting of 20 submaximal isometric unilateral plantar flexions. They were also instructed to complete the last two repetitions as hard and fast as possible. After five min of recovery, participants were subsequently tested for maximal voluntary contraction (MVC). Then, soleus M- and H-recruitment curves were constructed for all participants. Finally, nerve conduction measurements were performed as depicted in Fig. 1. Specifically, H-wave latencies and amplitudes were obtained in each testing condition (non-BFR vs. 60 vs. 80% BFR) at four different time points: [time point #1] non-restricted baseline, [time point #2] time control either with or without BFR, [time point #3] non-restricted pre-exercise, [time point #4] LI exercise either with or without BFR.

### Measurements

*Torque* All tests were performed unilaterally on the dominant lower limb while the participants remained comfortably seated on a Biodex System 3 Pro isokinetic dynamometer (Biodex Medical Systems, NY, United States) with their trunk, hips and testing thigh firmly strapped to an adjustable chair. The participants maintained their hips and knee flexed at 120° and ankle at 110° of plantar flexion^33^. The flexed position at the knee joint was chosen to reduce the mechanical contribution of the gastrocnemii to the plantar flexor torque^34^. The axis of rotation of the dynamometer was aligned with the anatomical ankle flexion-extension axis. During testing, all participants were asked to focus on the task and not to alter their posture.

*Surface EMG* Electrodes were placed following Surface for Electromyography for the Non-Invasive Assessment of Muscle (SENIAM) recommendations and surface EMG was recorded continuously from the soleus muscle during all testing sessions (Delsys DE 2.x series EMG sensors). To decrease the impedance of the interface between the skin and electrode, the skin was prepared by removing hair through skin abrasion and, then, cleaning with alcohol. A two-slot adhesive skin interface was applied on the EMG sensor to firmly stick the sensor to the skin. Once the appropriate electrode placements were confirmed, these locations were marked with indelible ink to ensure consistency for future test sessions. A conductive reference electrode was placed around the ankle of each participant. The surface EMG signals were band-pass filtered (20-450 Hz) and amplified using a Delsys Bagnoli-8 amplifier to a total gain of 1000. Then, a 16-bit analog-to-digital converter (National Instruments, USB-6251, TX, United States) was used to sample the signal at 10 kHz. EMG data were recorded in synchrony with the torque signal originating from the Biodex System using Mr. Kick software (Knud Larsen, SMI, Aalborg University, Denmark).

*Electrical stimulation* The posterior tibial nerve was stimulated with a constant-current an isolated stimulator (STIMSOLA, Biopac Systems, Inc., CA, United States). The self-adhesive cathode (8 mm diameter, Ag-AgCl) was located in the popliteal fossa and the anode (5 ×10 Compex, Medical SA) was placed proximal to the patella. Each participant was initially familiarized with a range of electrical stimuli (1–40 mA) over a period of ~five min. Before defining the final placement of the cathode electrode, the optimal position was identified using a handheld cathode ball electrode (0.5-cm diameter). Once the position eliciting the greatest response with the minimum stimulus intensity was determined, the stimulation electrode was firmly fixed to this site with rigid straps and taping.

*Resting arterial occlusion pressure* As in past research^35^, all blood flow measurements were taken during seated rest, mimicking the body position during exercise (e.g. seated plantar flexion). Arterial blood flow was detected using a vascular Doppler probe (SONOLINE B LCD Fetal Doppler 8 MHz vascular probe, CONTEC, China), placed over the posterior tibial artery, at the ankle level. Pulse was detected via auditory and visual signals obtained from the Doppler probe. A 13 ×124 cm pneumatic cuff (SC12L Tourniquet Cuffs, D. E. Hokanson, Inc., WA, United States) was placed on the most proximal end of the testing thigh and inflated using a rapid inflation device (E20 Rapid Cuff Inflator, D.E. Hokanson, Inc., WA, United States). The cuff was initially inflated to 50% of the individual resting systolic blood pressure and then raised gradually up to the point when tibial pulse was interrupted^27^. AOP was recorded as the nearest one mmHg pressure at which pulse was not present (AOP mean value: 150.7 ± 9.0 mmHg). AOP difference between both testing sessions was <5%, thus indicating that AOP was highly reproducible.

### Experimental protocols

*Muscle strength* MVC was recorded as the highest peak torque (N.m) obtained in response to three isometric contractions lasting five seconds each. One min of recovery was allowed between trials. Participants were instructed to exert their maximum force as hard as possible. Verbal encouragement, as well as audible and visual feedback from the dynamometer software, were provided to each participant. The highest plantar flexion MVC in each testing session was used to compute the submaximal target torque levels.

**Figure 2:**
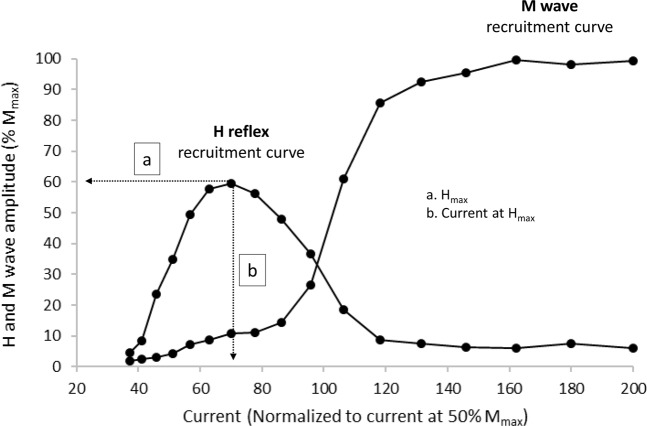
H- and M-wave recruitment curves for a representative participant. The following parameters were analyzed: (**a**) maximum amplitude of the H-reflex (H_max_) and (**b**) current intensity at H_max_.

*M- and H-wave recruitment curves* The M wave and H reflex were elicited at the beginning of each testing session while each participant maintained a low-level tonic contraction of the plantar flexors (10% MVC). This was done because reflex depression with a higher stimulus rate is greatly attenuated during a voluntary tonic contraction^36^. Participants were provided with online visual feedback of the torque exerted, which was displayed on a computer monitor. Testing procedures and recordings started by progressively increasing the current intensity by 5 mA from 0 until there was no further increase in peak twitch torque, nor in concomitant peak-to-peak M-wave amplitude. Three 1-ms rectangular pulses of were delivered at each intensity level. Then, at each current intensity, the preceding M-wave peak-to-peak amplitude was compared with the subsequent M-wave peak-to-peak amplitude. Once the preceding and subsequent M-wave peak-to-peak amplitude reached a plateau over the 3 stimulations, the current intensity of the previous stimulation was defined as the maximum current intensity. Then, to construct the M- and H-recruitment curves, the upper intensity was divided into 22 segments that were equally separated on a logarithmic scale^33,37^. For each current intensity, three stimuli were delivered at 3-s intervals. Figure 2 depicts further details concerning M- and H recruitment curves obtained at time point #1 in a representative participant.

*Nerve conduction* M-wave latencies were obtained from the sweeps corresponding to the maximal peak-to-peak amplitude of the M-recruitment curve. H-wave latencies were determined at current intensities compatible with the maximum peak-to-peak amplitude of this specific waveform on an individual basis. Figure 1 provides a schematic representation of the experimental protocol. The arrows signal the time points at which the latency and amplitude of H wave was obtained (immediately before and after each experimental period). Sixteen stimuli (at the previously determined H_max_ intensity) were delivered at a time interval of three seconds, while the participants sustained a continuous plantar flexion corresponding to 10% MVC. In the non-BFR condition, the stimulation was performed without BFR at all-time points. Conversely, in both BFR conditions (60 and 80% BFR), the stimulation was superimposed on BFR at the second and fourth time points.

*Blood-flow restriction with and without exercise* In line with past research, resistance exercise was prescribed for 75 repetitions, divided along four sets (30:15:15:15), with 30 s of rest between sets^5,12,26,38–42^. Participants performed isometric plantar flexions and each repetition involved two seconds of contraction and one second of relaxation (20% of MVC). Metronomic pacing ensured that all participants respected the cadence of isometric muscle contractions in both testing conditions. BFR pressure was set to 60 or 80% AOP in each session because there is compelling evidence that these levels of BFR are particularly effective for enhancing muscle activation during LI exercise^26^. BFR was achieved by inflating a nylon cuff (similar to that described for resting AOP measurements) within the most proximal portion of the thigh (inguinal fold region). Initially, the cuff was inflated to 3 progressive accommodative pressures (25, 50 and 75% of the individual cuff pressure corresponding to 60 or 80% BFR) during 30 s with 10 s of rest between them. Then, cuff pressure was adjusted to the final target value and sustained during the entire duration of the LI BFR exercise protocol (~5 min, including interset recovery periods). The exact same protocol was used to explore the impact of BFR alone on nerve conduction. However, in this case, the 5 min of continuous BFR were not accompanied by LI exercise.

### Data analysis

**Figure 3:**
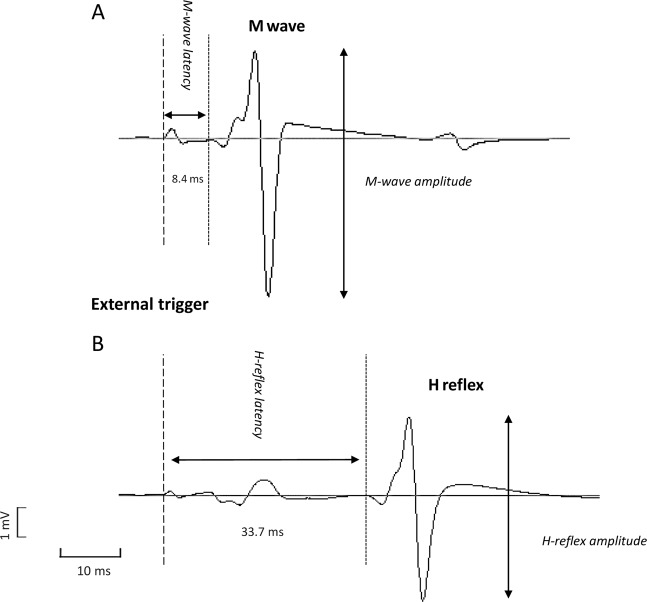
Representative example of M- and H-wave recorded from the soleus muscle at non-restricted baseline time point. The dotted vertical lines indicate the onset of M- and H wave and the double arrow represent the amplitude of each evoked potential. The latencies of each waveform are also included in the figure.

Nerve conduction was defined as the primary dependent variable. We therefore explored changes in electrophysiological properties of the sciatic-tibial nerve resulting from 3 conditions (non-BFR vs. 60 vs. 80% BFR), with and without exercise. Nerve conduction was estimated using the difference between the latency (in ms) of H and M wave (Fig. 3)^17^. The latency was considered as the time interval between the onset of the external trigger up to the first deflection of the H or M wave^18^. The peak-to-peak amplitude of H wave was also computed at each time point in all conditions. The amplitude of H wave obtained at each time point in all 3 conditions was normalized to the amplitude of M_max_. All 16 sweeps (1/stimulus) obtained in each time point were visually inspected. Then, the mean ± SD of the difference between the latency of both waveforms was calculated for each time point and the sweeps containing a variation >2 SD were eliminated from the final grand mean calculation.

### Statistical analysis

All data were tested for normality and homoscedasticity with the Kolmogorov–Smirnov and Mauchly’s test, respectively. Standard descriptive statistics were used to summarize the data. Based on a pilot study, including 6 participants, the difference between the H- and M-wave latencies corresponds to 24.7 ± 1.6 ms post-5 min of seated rest without BFR. After completing LI BFR exercise (BFR at 80% AOP), its values increase to 25.2 ± 1.5 ms. If the difference between these values represents the true difference between conditions, a sample size of 13 participants was estimated to have more than 80% power of correctly rejecting the null hypothesis. All measurements were taken as described in the methods section.

A one-way ANOVA was computed to explore whether the participants attained similar values for MVC, M_max_ peak-to-peak amplitude and M-wave latency between visits. A two-way ANOVA with repeated measures was used to test for the effect of condition (non-BFR vs. 60 vs. 80% BFR) and time (non-restricted baseline vs. time control either with or without BFR vs. non-restricted pre-exercise vs. LI exercise either with or without BFR) and condition-by-time interaction. When significant effects were detected (p < 0.05), post hoc *t* tests were used to locate the origin of the differences. We used Bonferroni’s adjustment for all repeated measures analyses. The eta-squared values (proportion of total variance that is attributable to an effect) are reported to indicate effect sizes for significant findings. Finally, in each condition, we calculated the delta values (and 95% confidence interval) for the difference between H and M wave latency in transition between different time points. This was done to obtain a graphical representation of the variability of the change in this specific parameter over time. All statistical analysis were computed using SPSS (version 24.0, SPSS Inc., IL, United States) and significance was set at p < 0.05. All data are reported as means ± SD.

### Ethical approval

All procedures performed in studies involving human participants were in accordance with the ethical standards of the institutional and/or national research committee (CEFMH N° 4/2017) and with the 1964 Helsinki declaration and its later amendments or comparable ethical standards.

## Results

Participants attained similar peak torque in all conditions (non-BFR: 71.1 ± 13.3, 60% BFR: 71.9 ± 13.3, 80% BFR: 74.6 ± 14.5 N.m; condition main effect: F_[2, 24]_ = 0.6, p = 0.56). The maximal peak-to-peak amplitude of M wave was also similar between conditions (non-BFR: 5.2 ± 2.5, 60% BFR: 4.9 ± 2.5, 80% BFR: 5.6 ± 2.6 mV; condition main effect: F_[2, 24]_ = 0.4, p = 0.38). No differences were observed between conditions for the latency of M_max_ response (non-BFR: 6.8 ± 0.9, 60% BFR: 6.3 ± 0.6, 80% BFR: 6.6 ± 0.8 ms; condition main effect: F_[2, 24]_ = 2.2, p = 0.13). Importantly, none of the participants experienced paresthesia or numbness during the course of the experiments.

Table 1 shows the absolute and normalized values of H-wave peak-to-peak amplitude at different time points in each condition (non-BFR, 60 and 80% BFR) with and without LI exercise. When compared to that seen in the non-BFR condition, BFR (adjusted to 60 or 80% BFR) did not affect the absolute amplitude of H wave, either with or without exercise (condition main effect: F_[2, 24]_ = 0.7, p = 0.48; time main effect: F _[3,36]_ = 0.9, p = 0.41; condition-by-time interaction: F_[6, 72]_ = 1.1, p = 0.36). Similar findings were also obtained for H-wave normalized amplitude (condition main effect: F _[2, 24]_ = 0.2, p = 0.84; time main effect: F_[3,36]_ = 0.3, p = 0.83; condition-by-time interaction: F_[6, 72]_ = 0.5, p = 0.72).

**Table 1:** Nerve conduction variables obtained with and without blood flow restriction (BFR) before and immediately after low-intensity (LI) exercise.

		Amp H (mV)	Amp H (%M_max_)	Lat H (ms)*	Lat H – Lat M (ms)*
Non-BFR	Baseline	2.0 ± 1.0	39.4 ± 13.0	32.7 ± 2.3	25.8 ± 2.3
	Non-BFR	1.8 ± 0.8	39.5 ± 18.4	33.3 ± 2.2	26.5 ± 2.3
	Pre-LI	1.4 ± 0.6	33.7 ± 19.4	33.6 ± 2.2	26.7 ± 2.3
	LI	1.6 ± 0.7	35.8 ± 16.3	33.4 ± 2.2	26.6 ± 2.4
60% BFR	Baseline	1.9 ± 0.9	39.8 ± 16.5	31.4 ± 1.8	25.0 ± 1.9
	BFR	1.8 ± 1.2	39.3 ± 21.1	31.7 ± 1.7	25.4 ± 1.9
	Pre-LIBFR	1.7 ± 1.0	37.6 ± 16.1	31.8 ± 1.7	25.5 ± 1.9
	LI BFR	1.7 ± 0.9	38.3 ± 19.7	31.9 ± 1.8	25.6 ± 2.0
80% BFR	Baseline	2.2 ± 1.5	37.6 ± 14.9	32.1 ± 1.5	25.4 ± 1.2
	BFR	1.9 ± 1.5	32.5 ± 14.9	32.7 ± 1.7	26.1 ± 1.5
	Pre-LIBFR	2.0 ± 1.2	35.5 ± 14.2	32.4 ± 1.4	25.8 ± 1.1
	LI BFR	2.3 ± 1.7	38.8 ± 18.5	32.6 ± 1.5	26.1 ± 1.2

Values are mean ± SD. **Abbreviations**: Amp H, H-reflex peak-to-peak amplitude; Lat H, H-wave latency; Lat H – Lat M, difference between the latency of H and M wave; BFR, blood flow restriction; LI, low-intensity exercise; LI BFR, low-intensity exercise with blood flow restriction. *Time main effect, p < 0.05.

The effect of each condition on H-wave latency at each time point, both with and without LI exercise, is also depicted in Table 1. As can be seen, there was a time main effect for H-wave latency (F_[3, 36]_ = 10.6; p < 0.0001, effect size: = 0.47). Post hoc analyses indicated that the latency of H wave increased between the two initial time points (from baseline to time control either with or without BFR; p = 0.02). Thereafter, H-wave latency remained unchanged between subsequent time points in all conditions. Importantly, the magnitude of these changes over time was similar between conditions (condition-by-time interaction: F_[6, 72]_ = 0.7; p = 0.48). We also obtained a significant time main effect for the difference between the latency of H and M wave (F_[3, 36]_ = 10.7, p < 0.0001, effect size: 0.47). Similarly to that seen in H-wave latency, follow up *t* tests revealed that the latency difference between both waveforms increased from baseline to time point #2 (time control either with or without BFR) (p = 0.02) and then remained essentially stable over time in all conditions. Again, the magnitude of these changes over time was similar between conditions (condition-by-time interaction: F_[6, 72]_ = 0.7; p = 0.47). The variability of change in the difference between H and M wave latency in transition between different time points can be seen in Fig. 3.

## Discussion

The main purpose of this study was to explore whether the pressure exerted by the pneumatic cuff during BFR, at 60 and 80% AOP, might exert a negative impact on nerve conduction. The effects of BFR on nerve conduction, with and without exercise, were determined using the amplitude of H reflex as well as the difference between the latency of H and M wave, a mixed sensoriomotor index of conduction velocity reflecting the functioning of the tibial nerve. Our findings indicate that acute submaximal BFR, sustained for ~ 5 min, does not affect the amplitude nor latency of H wave. When compared to that seen in the non-BFR control condition, the difference between the latency of H and M wave was also unaltered by BFR. Importantly, this was sustained for BFR elicited at 60 as well as 80% and was unaffected by LI resistance exercise. Thus, in healthy young adults, submaximal BFR does not disturb nerve conduction at either resting or exercising conditions. It is highly likely that, under these circumstances (submaximal pressure, applied at the proximal thigh), cuff pressure is innocuous to the integrity of the sciatic nerve because of low mechanical compression and prevention of tissue ischemia.

Despite focusing on an acute paradigm, our results are consonant with those of past research showing that LI BFR training does not affect H-wave latency of the exercised limb^11^. A different outcome was hypothesized because, in our experimental design, assessments of nerve conduction were performed while the lower limb of each participant remained exposed to the effects of BFR. Additionally, instead of using H-wave latency alone to estimate nerve conduction velocity, as was done in that study, we used the difference between H- and M-wave latencies. Calculations based on this approach are advantageous because they reflect the conduction time in the segment of the reflex arc with the start point from the popliteal fossa (Ia afferent) and the endpoint again at the popliteal fossa (α motoneuron). While the direct H-wave latency is affected by the additional time it takes for acetylcholine to transverse the synapse, to attach to receptors, and to generate muscle fiber action potential, this is not the case with the difference between the latency of H and M wave^43^. Moreover, using the time difference between the latencies of these waveforms, instead of the simple H-wave latency, is more suited for comparisons of nerve conduction between different conditions and times. This ensures that random changes in H-wave latency, as seen in several determinations in the same participant, are corrected by similar dispersion in the M wave latency^17^.

We found that acute BFR does not reduce H-wave amplitude. Importantly, this was extensive to both conditions (60 and 80% BFR) and occurred similarly with and without exercise. Since a decrease in evoked response amplitudes is typically seen in axonal function degeneration (e.g. neuropathy), our data suggest that, when prescribed within this spectrum of intensities, BFR does not trigger an acute axonal insult^44^. Recent research, concerning the effects of 4 weeks of LI BFR training on the amplitude of the soleus muscle H reflex, also demonstrated no chronic impact of this intervention on the maximal amplitude of H or M wave^12^. Importantly, in that investigation, BFR was elicited using a 13-cm-wide cuff and set according to the participants’ thigh circumference. Despite being different from adjusting BFR to a given % of AOP, this methodology is well known to be compatible with partial vascular restriction and has also been well validated in the available literature^45^.

In a previous report, it was shown that frequent low-load ischemic resistance training tends to disturb the EMG amplitude of the vastus medialis and attenuates EMG the amplitude of the rectus femoris muscle^10^. Despite suggesting an increased risk of neurapraxia with LI BFR exercise, unfortunately, these findings are difficult to compare with our results for two main reasons. First, instead of partial BFR, an ischemic stimulus was used in combination with LI exercise. As it is well known, ischemia is compatible with the inexcitability of peripheral axons (both sensory and motor) due to a decrease in persistent Na^+^ conductance during continuing depolarization^14^. Second, the vastus medialis and rectus femoris muscles are both innervated by the posterior division of the femoral nerve^46^. In the thigh, the femoral nerve lies in a groove between the iliacus and psoas major muscles, outside the femoral sheath and lateral to the femoral artery^46^. Therefore, compared to that seen in the sciatic nerve, the anatomical course of the femoral nerve is particularly superficial at the inguinal region^47^. This implicates a differential exposure to cuff pressure between both nerves (greater for the femoral nerve). For this reason, based on the available data (including that of the present study), it cannot be ascertained whether or not BFR, with or without exercise, exerts a negative effect on femoral nerve conduction. Accordingly, this needs to be systematically investigated in future research.

**Figure 4:**
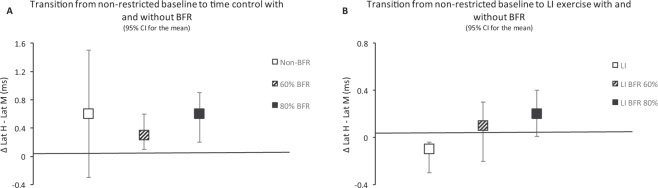
Delta difference between the latency of H- and M-wave (**A**) in transition from non-restricted baseline to time control with and without blood flow restriction (BFR), (**B**) in transition from non-restricted baseline to low-intensity (LI) exercise with and without BFR. Values are 95% confidence interval (CI) for the mean.

The difference between H and M-wave latency exhibited a small, but significant increase between the two initial time points (i.e. ~ 2% change from rest to time control with and without BFR). From then on, no further changes were noted in this specific parameter and this occurred similarly between control and BFR conditions. The variability of the change between time points can be seen in Fig. 4. The small increase in the difference between H- and M-wave latency at the initial stage of testing is likely secondary to physiological alterations induced by position-induced nerve strain. The selected testing position might have elicited tensile stress with parallel sciatic nerve excursion (i.e. elongation) at the level of the flexed hip^48^. Ultimately, this might have resulted in nerve strain followed by a reduction in intraneural blood flow and a subtle change in nerve conduction. This is supported by past research showing that hip flexion elongates the sciatic nerve bedding, causing strain, due to the posterior projection of the femoral head^49,50^. Unfortunately, since the latency of H reflex does not indicate whether a delayed evoked response is due to a disturbance in the motor, sensory, or both of these parts of the reflex pathway^51^, it is not possible to unravel the etiological basis of altered nerve conditions under these circumstances.

### Limitations

There is one important limitation to this study. Since M_max_ latency was only measured at non-restricted baseline in each condition, we cannot be sure that its latency of remained stable throughout the entire duration of the experimental protocol. Controlling for this effect would have implicated obtaining M-recruitment curves at each experimental time point (in addition to the sixteen stimuli delivered for H wave analysis). This would have prolonged the duration of BFR far beyond 5 min (the most widely used duration of BFR for neuromuscular adaptation), thus reducing the ecological validity of the present study. Moreover, it should be emphasized that sensory axons might be more vulnerable for ischemia than motor axons^14^. Therefore, we believe that our findings are not likely to be affected by this particular aspect. Finally, our findings cannot be extrapolated to people other than healthy young adults. Thus, the present data should not be interpreted as valid for establishing that BFR (with or without LI exercise) is free of potential neurological complications in the clinical populations.

## Conclusion

In conclusion, our findings indicate that neither the amplitude, nor latency of action potentials generated post-stimulation of the tibial nerve are affected by acute BFR, with or without LI exercise. Therefore, based on these data, and under the conditions used in this experimental design, the possibility of BFR raising the risk of sciatic nerve neuroapraxia is highly unlikely.

### Practical application

We provide preliminary evidence that performing LI exercise with BFR set at 60 or 80% AOP does not exert a negative impact on sciatic-tibial nerve function (unchanged amplitude and latency of evoked potentials). Thus, we believe that, from a neurological standpoint, LI BFR exercise may be regarded as a safe mode of resistance training in healthy young men.
